# Author Correction: A placental model of SARS-CoV-2 infection reveals ACE2-dependent susceptibility and differentiation impairment in syncytiotrophoblasts

**DOI:** 10.1038/s41556-023-01335-1

**Published:** 2023-12-18

**Authors:** J. Chen, J. A. Neil, J. P. Tan, R. Rudraraju, M. Mohenska, Y. B. Y. Sun, E. Walters, N. G. Bediaga, G. Sun, Y. Zhou, Y. Li, D. Drew, P. Pymm, W. H. Tham, Y. Wang, F. J. Rossello, G. Nie, X. Liu, K. Subbarao, J. M. Polo

**Affiliations:** 1 Department of Anatomy and Developmental Biology, Monash University, Clayton, Victoria Australia; 2 Development and Stem Cells Program, Monash Biomedicine Discovery Institute, Clayton, Victoria Australia; 3 Australian Regenerative Medicine Institute, Monash University, Clayton, Victoria Australia; 4 Department of Microbiology and Immunology, The University of Melbourne at the Peter Doherty Institute for Infection and Immunity, Melbourne, Victoria Australia; 5 Adelaide Centre for Epigenetics, Faculty of Health and Medical Sciences, The University of Adelaide, Adelaide, South Australia Australia; 6 South Australian Immunogenomics Cancer Institute, Faculty of Health and Medical Sciences, The University of Adelaide, Adelaide, South Australia Australia; 7 Implantation and Pregnancy Research Laboratory, School of Health and Biomedical Sciences, RMIT University, Melbourne, Victoria Australia; 8 Infectious Diseases and Immune Defences Division, The Walter and Eliza Hall Institute of Medical Research, Parkville, Victoria Australia; 9 Department of Medical Biology, University of Melbourne, Melbourne, Victoria Australia; 10 University of Melbourne Centre for Cancer Research, The University of Melbourne, Melbourne, Victoria Australia; 11 School of Life Sciences, Westlake University, Hangzhou, China; 12 Research Center for Industries of the Future, Westlake University, Hangzhou, China; 13 Westlake Laboratory of Life Sciences and Biomedicine, Hangzhou, China; 14 Westlake Institute for Advanced Study, Hangzhou, China; 15 WHO Collaborating Centre for Reference and Research on Influenza, Melbourne, Victoria Australia

**Keywords:** Stem-cell differentiation, Disease model, SARS-CoV-2

Correction to: *Nature Cell Biology* 10.1038/s41556-023-01182-0, published online 13 July 2023.

In the version of the article initially published, Y. Wang (RMIT University) was mistakenly omitted from the author list and contributions (derived the FT008 TSC line) and has now been added in the HTML and PDF versions of the article.

