# Supplementary material for: A placental model of SARS-CoV-2 infection reveals ACE2-dependent susceptibility and differentiation impairment in syncytiotrophoblasts
Source: Nat Cell Biol. 2023 Jul 13;25(8):1223–34. doi: 10.1038/s41556-023-01182-0 (PMC10415184; doi:10.1038/s41556-023-01182-0)
Supplement: Supplementary file 1 — Reporting Summary [file 41556_2023_1182_MOESM1_ESM.pdf]

nature portfolio

Corresponding author(s): Jose M. Polo, Kanta Subbarao

Last updated by author(s): 18 April 2023

Reporting Summary

Nature Portfolio wishes to improve the reproducibility of the work that we publish. This form provides structure for consistency and transparency in reporting. For further information on Nature Portfolio policies, see our Editorial Policies and the Editorial Policy Checklist.

Statistics

For all statistical analyses, confirm that the following items are present in the figure legend, table legend, main text, or Methods section.

n/a	Confirmed	
☐	☒	The exact sample size (*n*) for each experimental group/condition, given as a discrete number and unit of measurement
☐	☒	A statement on whether measurements were taken from distinct samples or whether the same sample was measured repeatedly
☐	☒	The statistical test(s) used AND whether they are one- or two-sided<br>*Only common tests should be described solely by name; describe more complex techniques in the Methods section.*
☐	☒	A description of all covariates tested
☒	☐	A description of any assumptions or corrections, such as tests of normality and adjustment for multiple comparisons
☐	☒	A full description of the statistical parameters including central tendency (e.g. means) or other basic estimates (e.g. regression coefficient) AND variation (e.g. standard deviation) or associated estimates of uncertainty (e.g. confidence intervals)
☐	☒	For null hypothesis testing, the test statistic (e.g. *F*, *t*, *r*) with confidence intervals, effect sizes, degrees of freedom and *P* value noted<br>*Give P values as exact values whenever suitable.*
☒	☐	For Bayesian analysis, information on the choice of priors and Markov chain Monte Carlo settings
☒	☐	For hierarchical and complex designs, identification of the appropriate level for tests and full reporting of outcomes
☒	☐	Estimates of effect sizes (e.g. Cohen's *d*, Pearson's *r*), indicating how they were calculated

Our web collection on statistics for biologists contains articles on many of the points above.

Software and code

Policy information about availability of computer code

Data collection	LightCycler 480 software (Roche, version 1.5.1.62), Leica application suite X version 3.7.1.21655 for DMi8, FLUOstar® Omega (BMG Labtech)
Data analysis	GraphPad Prism (v7), ImageJ (v1.8.0_112), Adobe Photoshop (version 21.2.3), base R(3.6.3), STAR aligner (v2.5.2), limma 3.42.2, tidyverse 1.3.0, trimmomatic 0.36, sabre 1.000, Subread v1.5.2, EdgeR v3.32.1, MADE4 v1.64.0, ComplexHeatmap v2.6.2, ggplot2 v3.3.5, ggrepel v0.9.1, Seurat v4.2.1, Monocle3 v1.3.1, dplyr v1.0.10, EdgeR v3.40.0, sva packagae v3.46

For manuscripts utilizing custom algorithms or software that are central to the research but not yet described in published literature, software must be made available to editors and reviewers. We strongly encourage code deposition in a community repository (e.g. GitHub). See the Nature Portfolio guidelines for submitting code & software for further information.

Data

Policy information about availability of data

All manuscripts must include a data availability statement. This statement should provide the following information, where applicable:

- Accession codes, unique identifiers, or web links for publicly available datasets
- A description of any restrictions on data availability
- For clinical datasets or third party data, please ensure that the statement adheres to our policy

Raw and processed next generation sequencing datasets were deposited at the Gene Expression Omnibus (GEO) repository with the following accession number: GSE185471

Human research participants

Policy information about studies involving human research participants and Sex and Gender in Research.

Reporting on sex and gender	This study utilised placental tissues obtained from pregnant women, so participants were females only, and sex and gender analysis does not apply to this study.
Population characteristics	Nothing to add here
Recruitment	Women undergoing elective termination of pregnancy were recruited, with exclusion criteria of known fetal abnormalities or participants <18 years of age. All participants provided informed written consent on placental tissue donation, and there is no self-selection bias or other biases that may affect the result.
Ethics oversight	Ethics approval for the use of first trimester human placental tissues for research was obtained from the Human Ethics Committee at Monash Health, Melbourne, Australia (RES-19-0000-399A).

Note that full information on the approval of the study protocol must also be provided in the manuscript.

Field-specific reporting

Please select the one below that is the best fit for your research. If you are not sure, read the appropriate sections before making your selection.

☒ Life sciences ☐ Behavioural & social sciences ☐ Ecological, evolutionary & environmental sciences

For a reference copy of the document with all sections, see nature.com/documents/nr-reporting-summary-flat.pdf

Life sciences study design

All studies must disclose on these points even when the disclosure is negative.

Sample size	We did not involve statistical methods to pre-determine the sample size, this was determined based on previous experience (Liu et al., Nature 2020) and other similar studies (Karvas et al., Cell Stem Cell 2022; Ruan et al., Cell Reports Medicine 2022). We generated data using 2 iTSC cell lines in multiple runs<br>of differentiation and infection (Representative graphs from 2 independent experiments showing n=3 separate infection replicates).
Data exclusions	No data were excluded.
Replication	Each experiment was repeated independently at least twice successfully, with each experiment containing a minimum of 3 separate infection replicates.
Randomization	Experiments were not randomized. Randomization of samples were not necessary to our study as we needed to determine virus vs control in most cases and our sample sizes were typically low.
Blinding	The investigators were not blinded during data collection and analysis, as neither human/animal studies or specific grouping were involved in this manuscript

Reporting for specific materials, systems and methods

We require information from authors about some types of materials, experimental systems and methods used in many studies. Here, indicate whether each material, system or method listed is relevant to your study. If you are not sure if a list item applies to your research, read the appropriate section before selecting a response.

Materials & experimental systems

n/a	Involved in the study
☐	☒ Antibodies
☐	☒ Eukaryotic cell lines
☒	☐ Palaeontology and archaeology
☒	☐ Animals and other organisms
☒	☐ Clinical data
☒	☐ Dual use research of concern

Methods

n/a	Involved in the study
☒	☐ ChIP-seq
☒	☐ Flow cytometry
☒	☐ MRI-based neuroimaging

Antibodies

Antibodies used

For Immunostaining:
Antibody, Company, Catalogue Number, Lot Number, Dilution:
Mouse anti-GATA2 IgG2a clone 2D11, Sigma-Aldrich Cat# WH0002624M1 Lot G2151-2D11, 1:100
Negative Control Rabbit Immunoglobulin Fraction, X0903, Dako, 1:10000
Mouse IgG1 Culture supernatant, X0931, Agilent Technologies, 1:10000
Goat Anti-Rabbit IgG Antibody (H+L), Biotinylated, BA-1000, Vector Laboratories, 1:1000
Horse Anti-Mouse IgG Antibody (H+L), Biotinylated, BA-2000, Vector Laboratories, 1:1000
Mouse anti-HCG IgG1 clone 5H4-E2, abcam , ab9582 Lot GR3285169-1, 1:200
Mouse anti-dsRNA IgG2a clone rJ2, Merck, MABE1134 Lot 3543801, 1:200
Rabbit anti-ACE2 IgG, abcam, ab15348 Lot GR3333640-15, 1:200
Mouse anti-GATA3 IgG1 clone 1A12-1D9, Invitrogen, MA1-028, 1:100
anti-HLA G IgG1 clone MEM-G/1, abcam, ab7759 Lot GR3262011-5, 1:50
Rabbit anti-SDC1 IgG, Cell Signaling Technology, Cat# 12922 Lot 1, 1:400
Rabbit anti-anti-DAB2 IgG, ab76253, abcam, 1:100
Rabbit anti-MMP2 IgG, Cell Signaling Technology, Cat# 40994, 1:100
Mouse anti-SARS-CoV-2 Nucleocapsid IgG, MyBioSource, MBS154642, 1:300
Goat anti-human IgG H&L (HRP), abcam, ab6858, 1:5000
Goat anti-mouse IgG1 Alexa Fluor 488, Invitrogen, Thermo Fisher Scientific, A21121 Lot 1964382, 1:400
Goat anti-rabbit IgG Alexa Fluor 555, Invitrogen, Thermo Fisher Scientific, A21428 Lot 1786491, 1:400
Goat anti-mouse IgG2a Alexa Fluor 555, Invitrogen, Thermo Fisher Scientific, A21137 Lot 1899521, 1:400
Donkey anti-rabbit IgG Alexa Fluor 647, Invitrogen, Thermo Fisher Scientific, A31573 Lot 1903516, 1:400
Donkey anti-mouse IgG Alexa Fluor 555, Invitrogen, Thermo Fisher Scientific, A31570, Lot 1850121, 1:400

For Western Blot:
Mouse Anti-GAPDH IgG1 clone 6C5, Merck, MAB374 Lot 3018865, 1:5000
Goat anti-Mouse IgG 680LT, LI-COR, 926-68020 Lot C20531-05, 1:50000
Goat anti-Rabbit IgG 800CW, LI-COR, 925-32211 Lot C80925-01, 1:50000

Validation

Antibodies obtained from th e commercial source were validated by the suppliers, detailed validation analysis relevant literatures are provided on the company website for the products used in this study. Some antibodies were validated in a previously published study as indicated in methods or relevant literature was cited.

GATA2 (WH0002624M1) https://www.sigmaaldrich.com/catalog/product/sigma/wh0002624m1
Negative Control Rabbit Immunoglobulin Fraction (X0903) https://www.agilent.com/cs/library/packageinsert/public/SSX0903RUO_01.pdf
Mouse IgG1 Culture supernatant (X0931) https://www.agilent.com/cs/library/packageinsert/public/102432002.PDF
Anti-Rabbit IgG Antibody (H+L), Biotinylated (BA-1000) https://vectorlabs.com/products/antibodies/biotinylated-goat-anti-rabbit-igg
Anti-Mouse IgG Antibody (H+L), Biotinylated (BA-2000) https://vectorlabs.com/products/antibodies/biotinylated-horse-anti-mouse-igg
HCG (ab9582) https://www.abcam.com/hcg-beta-antibody-5h4-e2-ab9582.html
dsRNA (MABE1134) https://www.merckmillipore.com/AU/en/product/Anti-dsRNA-Antibody-clone-rJ2,MM_NF-MABE1134-25UL
ACE2 (ab15348) https://www.abcam.com/ace2-antibody-ab15348.html
GATA3 (MA1-028) https://www.thermofisher.com/antibody/product/GATA3-Antibody-clone-1A12-1D9-Monoclonal/MA1-028
HLA G (ab7759) https://www.abcam.com/hla-g-antibody-mem-g1-ab7759.html
SDC1 (Cat# 12922) https://www.cellsignal.com/products/primary-antibodies/syndecan-1-d4y7h-rabbit-mab/12922
MMP2 (Cat# 40994) https://www.cellsignal.com/products/primary-antibodies/mmp-2-d4m2n-rabbit-mab/40994
SARS-CoV-2 Nucleocapsid (MBS154642) https://www.mybiosource.com/monoclonal-covid-19-antibody/covid-19-nucleocapsid-np-coronavirus/154642
Goat anti-Human IgG H&L (HRP) https://www.abcam.com/products/secondary-antibodies/goat-human-igg-hl-hrp-ab6858.html
Goat anti-mouse IgG1 Alexa Fluor 488 (A21121) https://www.thermofisher.com/antibody/product/Goat-anti-Mouse-IgG1-Cross-Adsorbed-Secondary-Antibody-Polyclonal/A-21121
Goat anti-rabbit IgG Alexa Fluor 555 (A21428) https://www.thermofisher.com/antibody/product/Goat-anti-Rabbit-IgG-H-L-Cross-Adsorbed-Secondary-Antibody-Polyclonal/A-21428
Goat anti-mouse IgG2a Alexa Fluor 555 (A21137) https://www.thermofisher.com/antibody/product/Goat-anti-Mouse-IgG2a-Cross-Adsorbed-Secondary-Antibody-Polyclonal/A-21137
Donkey anti-rabbit IgG Alexa Fluor 647 (A31573) https://www.thermofisher.com/antibody/product/Donkey-anti-Rabbit-IgG-H-L-Highly-Cross-Adsorbed-Secondary-Antibody-Polyclonal/A-31573
Donkey anti-mouse IgG Alexa Fluor 555 (A31570)
https://www.thermofisher.com/antibody/product/Donkey-anti-Mouse-IgG-H-L-Highly-Cross-Adsorbed-Secondary-Antibody-

Polyclonal/A-31570
GAPDH (MAB374) https://www.merckmillipore.com/AU/en/product/Anti-Glyceraldehyde-3-Phosphate-Dehydrogenase-Antibody-clone-6C5,MM_NF-MAB374
Goat anti-mouse IgG 680LT (926-68020) https://www.licor.com/bio/reagents/irdye-680lt-goat-anti-mouse-igg-secondary-antibody
Goat anti-rabbit IgG 800CW (925-32211) https://www.licor.com/bio/reagents/irdye-800cw-goat-anti-rabbit-igg-secondary-antibody

Eukaryotic cell lines

Policy information about cell lines and Sex and Gender in Research

Cell line source(s)	Human iTSC lines were previously generated as described in Liu et al., Nature 2020. (https://doi.org/10.1038/s41586-020-2734-6). iTSCs were generated using human fibroblasts sourced from ThermoFisher (Catalogue number, C-013-5C and lot#1528526 for 55F and lot#1569390 for 32F. Vero cells (Cat no. CCL-81) and Calu-3 cells (Cat no. HTB-55) were purchased from ATCC. Vero E6-TMPRSS2 (Cat no. JCRB1819) were purchased from CellBank Australia. Expi293 cells (Cat no. 14527) were purchased from Thermo Fisher Scientific. Ishikawa cells were sourced from Sigma-Aldrich (Catalogue number, 99040201, lot #14B013). FT008 primary cell lines were derived from the tissue consented by patients. However, as agreed in the initial ethics to receive the cells, the sex of the donor was not disclosed. However, we have analyzed sequencing data and found their genotype to be male.
Authentication	The human fibroblasts, Vero cells, Calu-3 cells, Vero E6-TMPRSS2, Expi293 and Ishikawa cells have been authenticated by the manufacturer's company via assays such as cellular morphology, STR profilling, mycoplasma testing, sterility testing or growth profile as stated in the certificate of analysis. The iTSCs and FT008 have been also authenticated in-house by immunostaining, qPCR, RNA-seq.
Mycoplasma contamination	Furthermore, all cell lines were regularly tested and were mycoplasma negative.
Commonly misidentified lines (See ICLAC register)	No commonly misidentified cell lines were used in this study.
