# Supplementary figures and images for: A placental model of SARS-CoV-2 infection reveals ACE2-dependent susceptibility and differentiation impairment in syncytiotrophoblasts
Source: Nat Cell Biol. 2023 Jul 13;25(8):1223–34. doi: 10.1038/s41556-023-01182-0 (PMC10415184; doi:10.1038/s41556-023-01182-0)

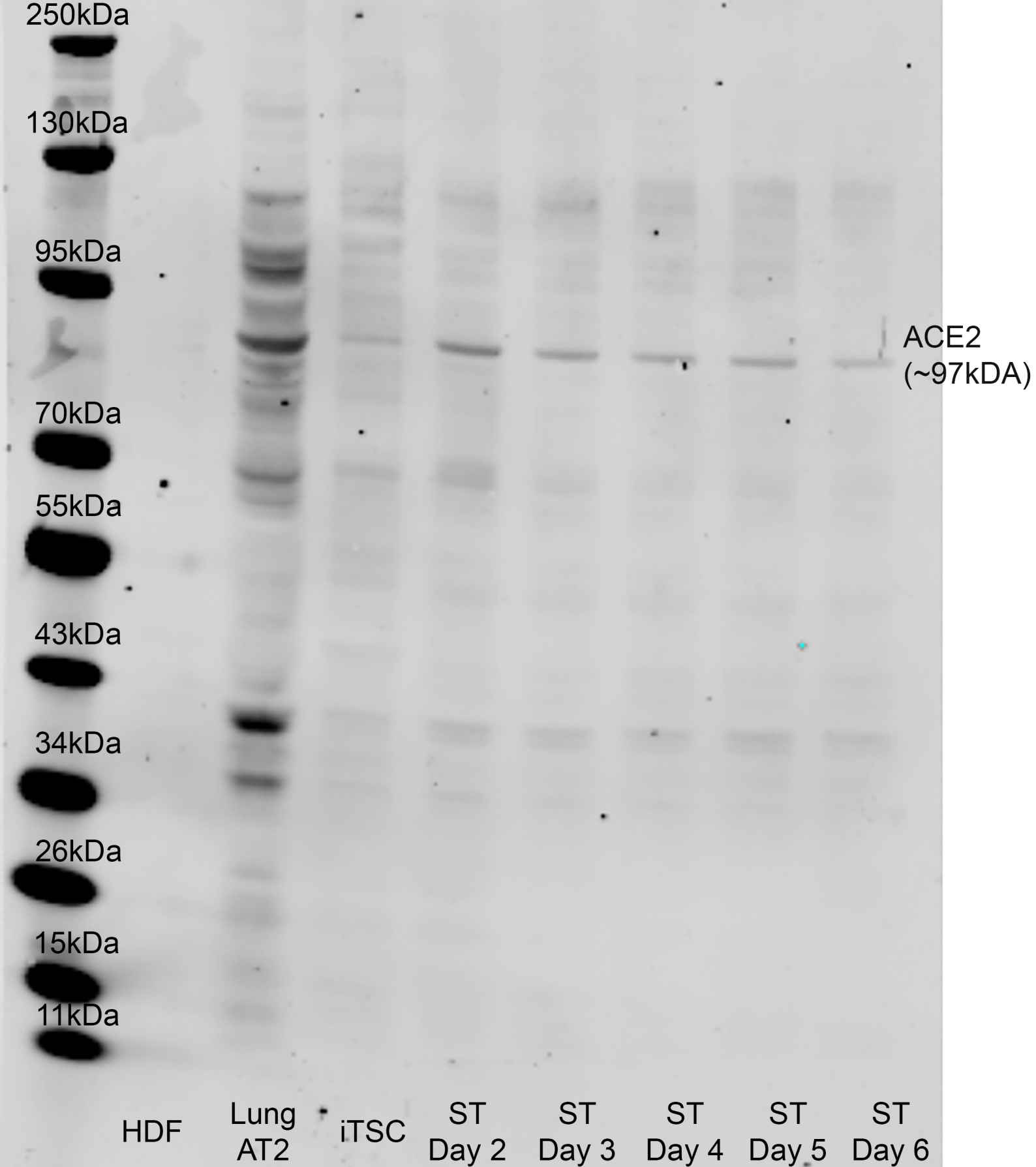

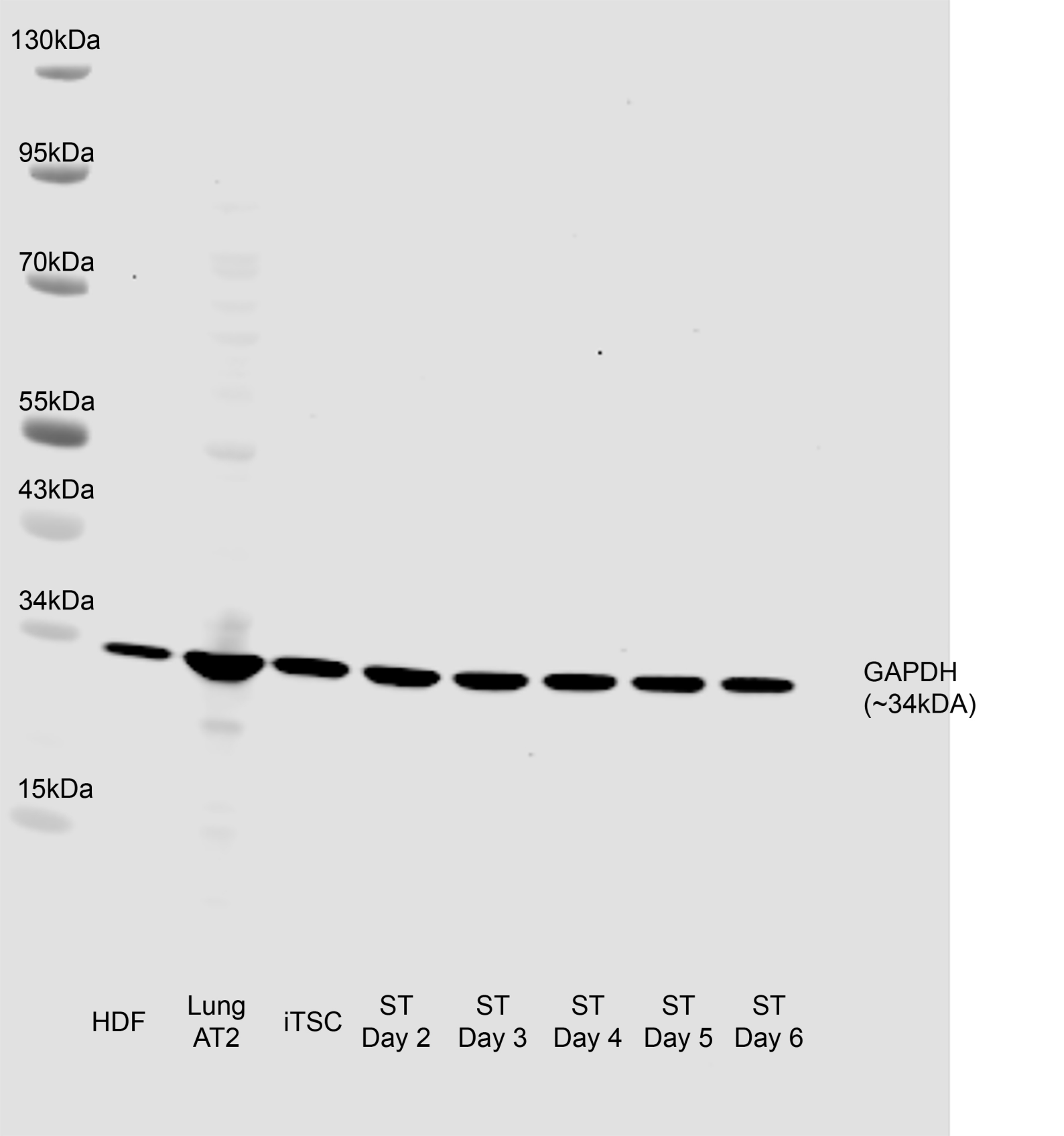

Supplement: Supplementary file 11 — Statistical source data for graphs. [file 41556_2023_1182_MOESM11_ESM.pdf]
